# Cytochrome P450 Inhibitors Reduce Creeping Bentgrass (*Agrostis stolonifera*) Tolerance to Topramezone

**DOI:** 10.1371/journal.pone.0130947

**Published:** 2015-07-17

**Authors:** Matthew T. Elmore, James T. Brosnan, Gregory R. Armel, Dean A. Kopsell, Michael D. Best, Thomas C. Mueller, John C. Sorochan

**Affiliations:** 1 Department of Soil and Crop Sciences, Texas A&M AgriLife Extension Service, Dallas, Texas, United States of America; 2 Department of Plant Sciences, The University of Tennessee, Knoxville, Tennessee, United States of America; 3 Department of Chemistry, The University of Tennessee, Knoxville, Tennessee, United States of America; University of Illinois at Urbana-Champaign, UNITED STATES

## Abstract

Creeping bentgrass (*Agrostis stolonifera* L.) is moderately tolerant to the *p*-hydroxyphenylpyruvate dioxygenase-inhibiting herbicide topramezone. However, the contribution of plant metabolism of topramezone to this tolerance is unknown. Experiments were conducted to determine if known cytochrome P450 monooxygenase inhibitors 1-aminobenzotriazole (ABT) and malathion alone or in combination with the herbicide safener cloquintocet-mexyl influence creeping bentgrass tolerance to topramezone. Creeping bentgrass in hydroponic culture was treated with ABT (70 μM), malathion (70 μm and 1000 g ha^-1^), or cloquintocet-mexyl (70 μM and 1000 g ha^-1^) prior to topramezone (8 g ha^-1^) application. Topramezone-induced injury to creeping bentgrass increased from 22% when applied alone to 79 and 41% when applied with malathion or ABT, respectively. Cloquintocet-mexyl (70 μM and 1000 g ha^-1^) reduced topramezone injury to 1% and increased creeping bentgrass biomass and PSII quantum yield. Cloquintocet-mexyl mitigated the synergistic effects of ABT more than those of malathion. The effects of malathion on topramezone injury were supported by creeping bentgrass biomass responses. Responses to ABT and malathion suggest that creeping bentgrass tolerance to topramezone is influenced by cytochrome P450-catalyzed metabolism. Future research should elucidate primary topramezone metabolites and determine the contribution of cytochrome P450 monooxygenases and glutathione *S*-transferases to metabolite formation in safened and non-safened creeping bentgrass.

## Introduction

Creeping bentgrass (*Agrostis stolonifera* L.) is the most widely used cool-season turfgrass on golf course fairways and tees in the United States [1]. Crabgrass (*Digitaria* spp.), goosegrass (*Eleusine indica* L. Gaertn.), and bermudagrass (*Cynodon* spp.) are difficult-to-control weeds that disrupt the functional and aesthetic quality of creeping bentgrass turf [2]. Herbicide options for weed control in creeping bentgrass are limited. Herbicides such as fenoxaprop-*p*-ethyl and quinclorac often cannot be applied at rates that provide acceptable weed control as they are too injurious to creeping bentgrass [3–8].

Topramezone is a pyrazolone *p*-hydroxyphenylpyruvate dioxygenase (HPPD; EC 1.13.11.27)-inhibiting herbicide registered for use in corn (*Zea mays* L.) and turfgrass [9,10]. When applied between 12 and 37 g ha^-1^, topramezone can control multi-tiller crabgrass and goosegrass, while sequential applications can suppress bermudagrass [11–13]. Topramezone is registered for application to most C_3_ turfgrass species at ≤ 37 g ha^-1^, but application to creeping bentgrass can cause injury at 6 to 37 g ha^-1^ [10,11,13,14]. However, if creeping bentgrass injury from topramezone could be reduced without sacrificing weed control, it would be an excellent tool for weed management.

Our previous research demonstrated that creeping bentgrass is more tolerant to topramezone than large crabgrass or goosegrass; the herbicide safener cloquintocet-mexyl further increases this tolerance and does not reduce topramezone efficacy against large crabgrass and goosegrass [15]. Desirable herbicide safeners protect graminaceous crop plants from herbicide injury more than target weeds, increasing the margin of selectivity [16]. Safeners function predominately by increasing activity of cytochrome P450 monooxygenases and transferases that catalyze phase I or II reactions involved in herbicide metabolism [16–21]. The safener cloquintocet-mexyl is used commercially in wheat to increase selectivity of acetyl-CoA carboxylase (EC 6.4.1.2) and acetolactate synthase (EC 2.2.1.6) inhibiting herbicides [22].

**Fig 1:**
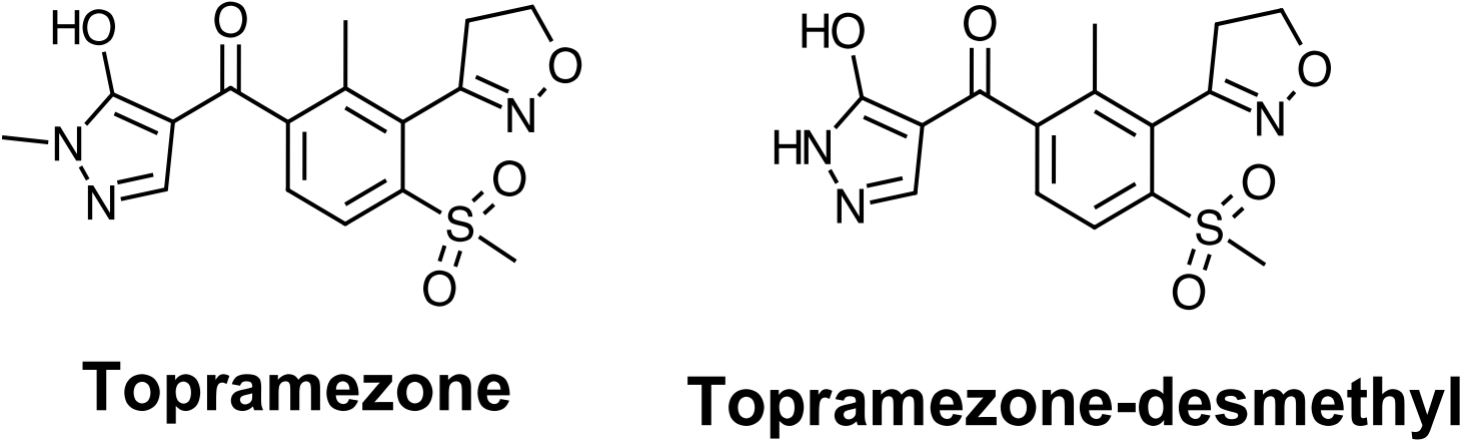
Topramezone and its primary metabolite, topramezone-desmethyl as proposed by Grossman and Ehrhardt (2007).

Excellent corn tolerance has been attributed to rapid N-demethylation of topramezone to an inactive metabolite, which is different than the rapid hydroxylation that provides corn tolerance to triketone HPPD-inhibitors such as mesotrione (Fig 1)[9]. A mutation to an allele coding for P450 enzymes in corn hybrids reduces tolerance to the mesotrione and tembotrione, especially if the hybrid is homozygous for the non-functional allele. While homozygous hybrids were injured >50% by mesotrione and tembotrione, topramezone caused no injury to any of the hybrids tested [23]. Corn tolerance to mesotrione and presumably tembotrione is dependent on a P450-mediated hydroxylation at the 4-position on the cyclohexandione ring of these triketone herbicides [24]. The observation that corn hybrids with non-functional P450 alleles are less injured by topramezone than mesotrione and topramezone suggests that a different metabolic mechanism or different P450 monooxygenases not coded for by the mutant allele may confer corn tolerance to topramezone. Grossman and Ehrhardt [9] demonstrated that differential affinity of topramezone to corn and grassy weed HPPD was not the main mechanism conferring corn tolerance, which suggests metabolism may be important. While tolerance mechanisms have been researched in corn, the constitutive and cloquintocet-induced mechanisms of creeping bentgrass tolerance to topramezone are unknown. Cloquintocet-mexyl can increase cytochrome P450 monooxygenase and glutathione *S*-transferase (GST) activity in wheat (*Triticum aestivum* L.) and has also been reported to increase pyridinyl N-glucosylation of clodinafop-propargyl [20, 25–27]. Cloquintocet-mexyl can also increase O-glycosyltransferase activity in wheat, and it is possible that topramezone metabolism may also proceed via O-glucosylation at the hydroxyl group [28].

Cytochrome P450 inhibitors such as 1-aminobenzotriazole (ABT), piperonyl butoxide (PBO), and malathion are commonly used to determine if P450-catalyzed reactions affect plant tolerance to herbicides [18,29]. Similar to topramezone, herbicides such as chlorotoluron and metflurazon can be N-demethylated to their primary metabolites. These N-demethylations are thought to be P450-catalyzed [30]. ABT can reduce metabolism and increase the toxicity of chlorotoluron to wheat and herbicide-resistant biotypes of *Lolium rigidum*, *Bromus tectorum* and *Alopecurus myosuroides* [31–33]. Interestingly, ABT synergizes chlorotuluron by inhibiting aryl ring alkyl hydroxylation, and does not affect N-demethylation [32,34]. While these research reports suggest that ABT does not inhibit N-demethylase activity, ABT can inhibit the N-demethylation of metflurazon to the herbicidally active norfluazon in unicellular green algae (*Chlorella fusca*) [35]. Similar to ABT, malathion can synergize acetolactate synthase (ALS; EC 2.2.1.6)-inhibiting and acetyl-CoA carboxylase (EC 6.4.1.2)-inhibiting herbicides that are metabolized via hydroxylation [36–40]. However, there are no reports on plant tolerance to topramezone in the presence of P450 inhibitors.

The objective of this research was to determine if P450 inhibitors affect creeping bentgrass tolerance to topramezone in the presence and absence of cloquintocet-mexyl. Our hypothesis was that P450-mediated metabolism of topramezone is a route of topramezone detoxification in creeping bentgrass. Thus, creeping bentgrass tolerance to topramezone would be reduced by the P450 inhibitors ABT and malathion. Our second hypothesis was that the effects of these P450 inhibitors would be offset by cloquintocet-mexyl, which can increase plant metabolism of herbicides through non-P450-catalyzed routes or through P450 monooxygenases not inhibited by ABT and malathion.

## Materials and Methods

### Plant material and growing conditions

Plants were grown and experiments were conducted in a glasshouse at the University of Tennessee (Knoxville, TN; 35° 53’ N lat.). Creeping bentgrass (*Agrostis stolonifera* L. c.v. ‘Penncross’) seeds were planted into cone-tainers (3.8 cm diameter 20 cm depth; Stuewe and Sons, OR, USA) filled with a peat moss, perlite, and vermiculite growing medium (Fafard No. 2, Sun Gro Horticulture, MA, USA). After germination, cone-tainers were hand-thinned to contain one plant each. Plants were fertilized monthly using a complete (20N:20P_2_O_5_:20K_2_O) (Howard Johnson’s Triple Twenty Plus Minors, WI, USA) fertilizer at 25 kg N ha^-1^, irrigated as needed to prevent wilt and maintained at a 2.5 cm height of cut with scissors twice weekly.

After six months of growth, roots were washed to completely remove the peat-based growth media and cut to a uniform 15 cm length to stimulate new root growth from the plant crown. The roots were then inserted through 7 mm holes in the lid of a 23 cm diameter polyethylene tub filled with 7 L of half-strength Hoagland nutrient solution [41]. Deionoized water was added as needed to maintain a 7 L volume throughout the experiment. The exterior of the tubs and lids were covered in silver paint to prevent algal growth in the nutrient solution. A blower fan was used to pump ambient air through Tygon (Saint-Gobain Performance Plastics, OH, USA) tubing and a 2 cm diameter spherical airstone (Rolf C. Hagen Corp., MA, USA) submerged in the nutrient solution. Each polyethylene tub comprised an experimental unit and contained three creeping bentgrass plants.

### Herbicide, P450 Inhibitor, and Safener Treatment

Creeping bentgrass remained in hydroponic culture in the absence of P450 inhibitors for 10 days. After this 10-day acclimation period, the cytochrome P450 inhibitors ABT (Technical grade, Alfa Aesar, USA) and malathion (50% EC, Spectrum Brands, USA), as well as the herbicide safener cloquintocet-mexyl (Technical grade, Bosche Scientific, USA) were added to the nutrient solution in appropriate amounts to achieve a 70 μM concentration 24 hours prior to topramezone application. Optimum absorption and translocation of ABT is thought to occur through plant roots [42]. Conversely, cloquintocet-mexyl and malathion penetrate leaf cuticles well and are usually applied to leaf tissue to determine their effects on herbicides [18, 42]. However, preliminary research determined that cloquintocet-mexyl added to nutrient solution reduced injury from topramezone more effectively than foliar applications. Therefore, in addition to their inclusion in nutrient solution, cloquintocet-mexyl and malathion were also applied to creeping bentgrass foliage at 28 and 1000 g ha^-1^, respectively, 22 hours after their addition to the nutrient solution (two hours prior to topramezone application). The cloquintocet-mexyl rate was based on our previous research and the malathion rate is commonly used in investigations to determine mechanisms of herbicide resistance [15,36].

The inhibitors and safener compounds were arranged into six treatments as follows: (1) ABT; (2) malathion; (3) cloquintocet-mexyl; (4) ABT + cloquintocet-mexyl; (5) malathion + cloquintocet-mexyl; and (6) non-treated control. These treatments were applied alone or in conjunction with topramezone (BAS 670H 2.8 SC; BASF Corp., USA) at 8 g ha^-1^ to creeping bentgrass foliage. Preliminary research suggested this topramezone dose would be sub-lethal. Treatments were arranged in a randomized complete block design with three replications and the experiment was repeated in time.

ABT, malathion, and cloquintocet-mexyl were dissolved in 7 mL dimethyl sulfoxide and added to 7 L of nutrient solution 24 hours before topramezone application. Dimethyl sulfoxide was also added to non-treated nutrient solutions. All compounds remained in solution after addition to the nutrient solution. This was expected as the water solubility of ABT (25 mg L^-1^), malathion (145 mg L^-1^) and cloquintocet-mexyl (> 590 mg L^-1^) of these compounds is greater than the amounts applied to achieve a 70 μM concentration. Additionally, the blower system maintained a low level of agitation in the nutrient solutions throughout the duration of the experiment. The ABT and malathion concentrations were based on the methods of previous research evaluating the effects of P450 inhibitors on chlorotoluron metabolism [42,43].

Foliar applications of topramezone, malathion, and cloquintocet-mexyl were all performed separately but in the same manner. All treatments were applied with non-ionic surfactant (Activator 90. Loveland Products Inc., USA) at 0.25% v/v in 215 L ha^-1^ of water carrier through a single flat-fan nozzle (8004 EVS; Spraying Systems Co., USA) in a spray chamber (Generation III Research Track Sprayer. DeVries Manufacturing, USA). To prevent runoff of the spray solution from the polyethylene lid into the nutrient solution, a temporary paper backing lined with plastic was fitted between the creeping bentgrass foliage and lid and was removed after spray applications.

### Evaluating Treatment Effects

Before visible symptoms of topramezone injury were observed, photosystem II (PS II) maximum quantum photosynthetic yield (F_v_/F_m_) was assessed 2 days after treatment (DAT). Other researchers [44–45] have used F_v_/F_m_ for a quantitative measurement of the effects of HPPD-inhibiting herbicides, as it is correlated to reductions in chlorophyll and carotenoid concentrations; these reductions are an indirect, but consequential mechanism of HPPD-inhibition [46]. F_v_/F_m_ was measured using a pulse-modulated fluorometer (OS1-FL, Opti-sciences, Inc., USA) by subtracting F_o_, the minimal level of fluorescence, from F_m_, the maximum level of fluorescence and dividing this difference by F_m_ [46]. Four measurements on each of the three plants in each experimental unit comprised of 12 subsamples that were averaged to determine a mean value.

Visible injury of creeping bentgrass leaf tissue was evaluated at 7 and 10 DAT on a 0 (no bleaching or necrosis) to 100% (complete leaf bleaching or necrosis) scale. Only data collected 10 DAT will be presented, as treatment responses were most apparent at this time.

Biomass was also collected 10 DAT. Scissors were used to remove all stem, leaf, and stolon tissue. The harvested tissue from all three plants in each experimental unit was combined and placed in a drying oven at 80°C for 48 h and weighed. Biomass data were transformed to a percentage of the non-treated control to determine treatment effects on overall plant vigor.

### Statistical Analysis

Treatments were arranged in a single-factor randomized complete block design with three replications. Plants that were treated with P450 inhibitors or cloquintocet, but not topramezone were analyzed separately and compared to the non-treated control. The non-treated control was included in the analysis for F_v_/F_m_ and biomass of plants treated with topramezone but removed for analysis of visible injury. Model assumptions were tested through residual analysis (Shapiro–Wilk statistic) in SAS (Statistical Analysis Software, Inc., Cary, NC), and no transformations were needed. ANOVA, mean separations, and contrasts were conducted in SAS with main effects and all possible interactions tested using the appropriate expected mean square values as described by McIntosh [47]. Fisher’s protected LSD (P ≤ 0.05) was used to separate means.

## Results

Treatment interactions with experimental run were not detected (P ≤ 0.05); therefore, data were combined across runs. For plants treated with topramezone, safener and P450 inhibitor treatments had a significant effect on F_v_/F_m_, biomass, and visible injury (P ≤ 0.01).

### PSII quantum yield (F_v_/F_m_) responses

**Fig 2:**
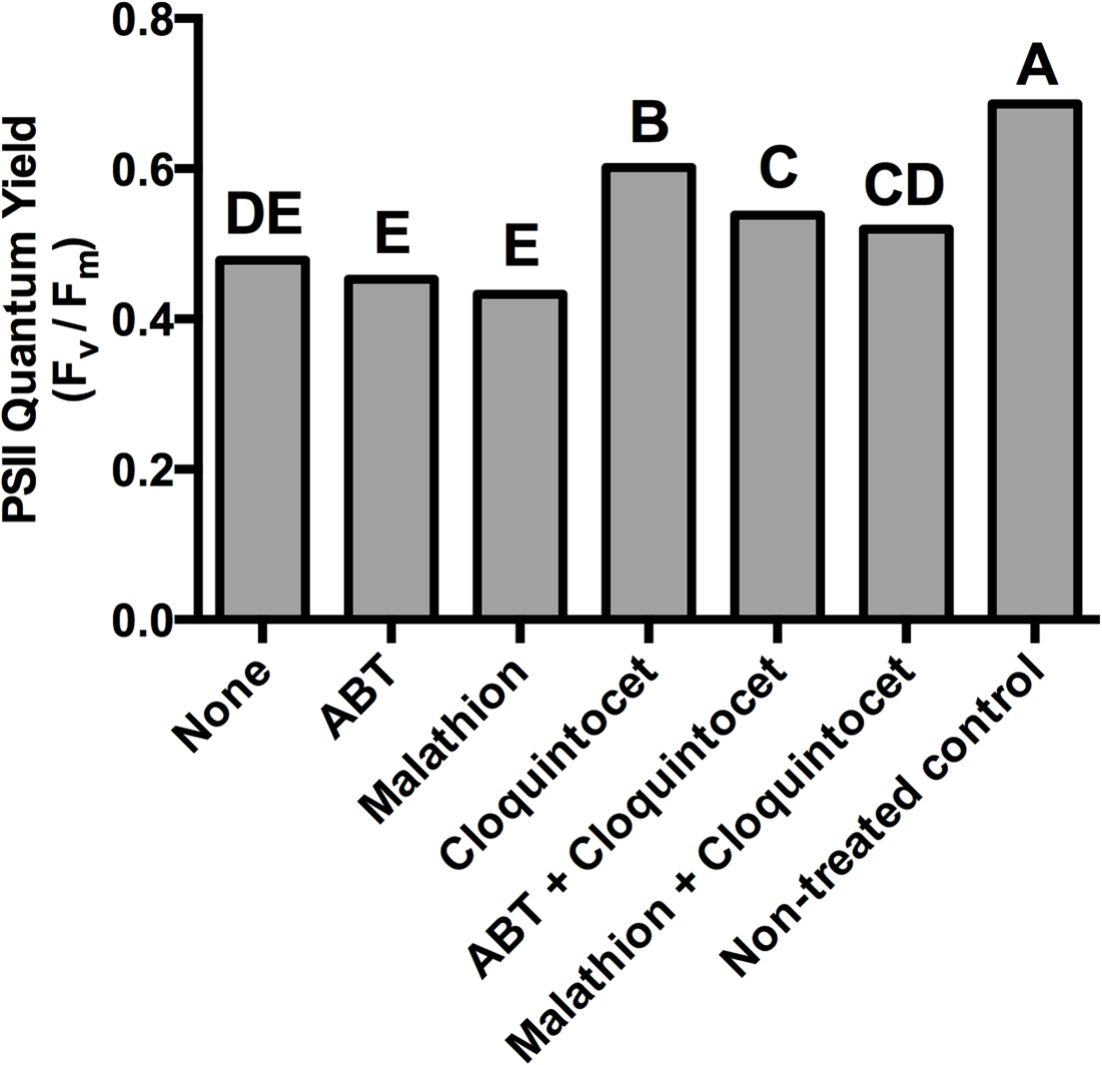
PS II maximum quantum yield (F_v_/F_m_) of creeping bentgrass leaf tissue two days after treatment. Plants were treated with topramezone (8 g ha^-1^) alone or in combination with the cytochrome P450 monooxygenase inhibitors 1-aminobenzotriazole (ABT) or malathion. Creeping bentgrass was also treated with the herbicide safener cloquintocet-mexyl (cloquintocet) alone and in combination with ABT or malathion. A non-herbicide treated control is included for comparison. Columns containing the same letter are not statistically different as determined by Fisher’s Protected LSD Test (α ≤ 0.05).

Malathion, ABT, cloquintocet-mexyl, or combinations thereof did not affect F_v_/F_m_ in the absence of topramezone (data not presented). This is similar to results of previous research [34,42]. Regardless of P450 inhibitor or safener inclusion, topramezone reduced the F_v_/F_m_ compared to the non-treated control (Fig 2). F_v_/F_m_ values are similar to those reported in previous research evaluating topramezone against hybrid bermudagrass at 5 DAT. When applied alone or to plants treated with ABT or malathion, topramezone reduced the F_v_/F_m_ similarly by > 25%. Among plants treated with topramezone, those also treated with cloquintocet-mexyl had a higher F_v_/F_m_ than others. Plants treated with cloquintocet-mexyl alone had a higher F_v_/F_m_ than those treated with the combination of cloquintocet-mexyl and malathion or ABT. Visible injury symptoms were not apparent at 2 days after treatment when F_v_/F_m_ was measured.

### Visible responses 10 days after treatment

**Fig 3:**
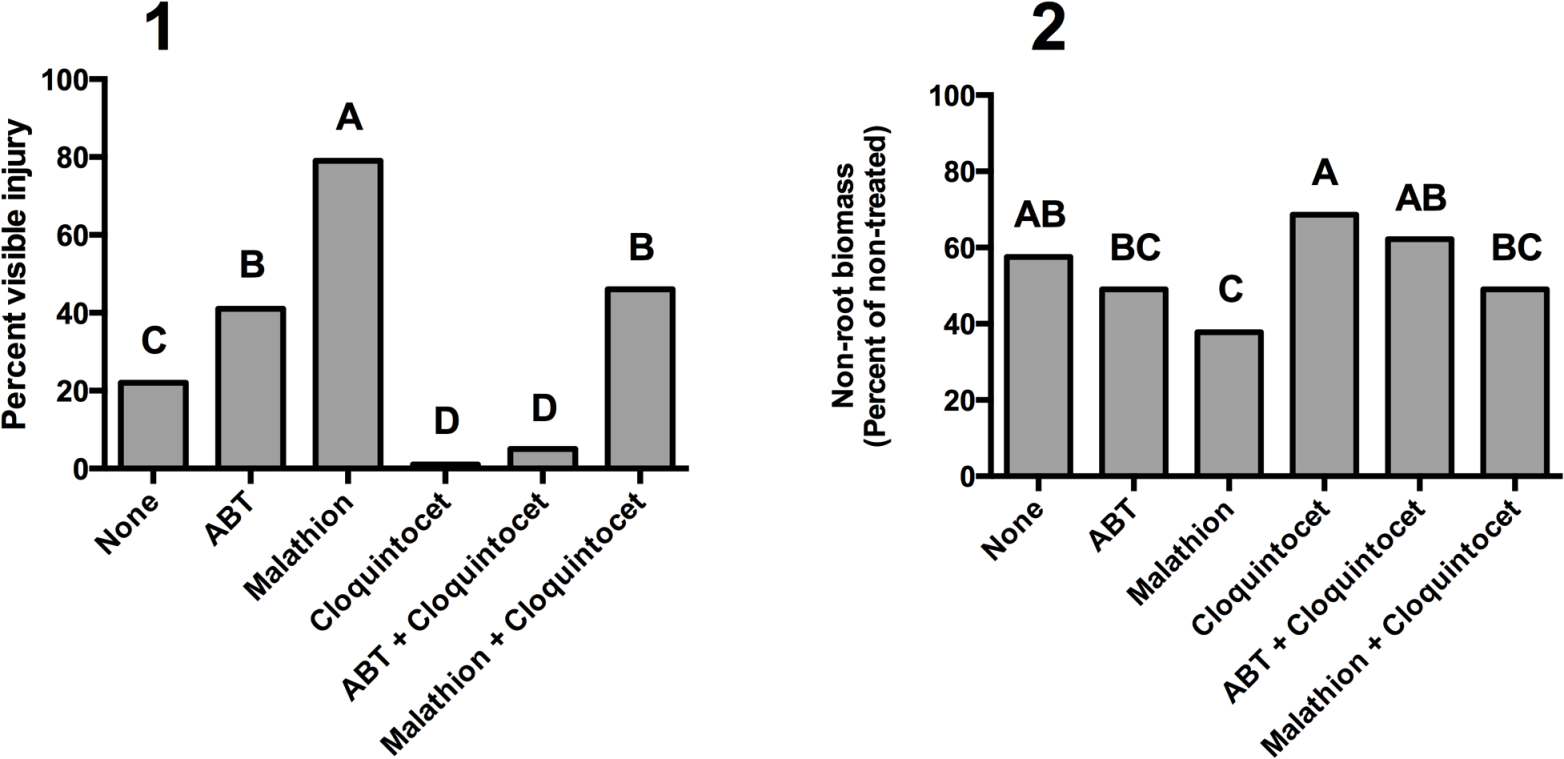
Visible injury (1) and biomass (2) of creeping bentgrass leaf tissue 10 days after treatment. Plants were treated with topramezone (8 g ha^-1^) alone or in combination with the cytochrome P450 monooxygenase inhibitors 1-aminobenzotriazole (ABT) or malathion. Creeping bentgrass was also treated with the herbicide safener cloquintocet-mexyl (cloquintocet) alone and in combination with ABT or malathion. A non-herbicide treated control is included for comparison. Visible injury of creeping bentgrass leaf tissue was evaluated on a 0 (no bleaching or necrosis) to 100% (complete leaf bleaching or necrosis) scale. Columns containing the same letter are not statistically different as determined by Fisher’s Protected LSD Test (α ≤ 0.05).

Topramezone applied alone caused 22% visible injury, mostly in the form of bleaching, which is characteristic of HPPD-inhibiting herbicide applications (Fig 3). Malathion increased injury from topramezone to 79%, more than observed from any other treatment. Both visible bleaching and bleaching-induced necrosis were observed on plants treated with malathion. ABT also increased topramezone injury to 41%, but injury was less than that observed with malathion. Plants treated with cloquintocet-mexyl alone displayed only 1% injury, which was similar to that of the ABT and cloquintocet-mexyl combination. Reductions in visible bleaching from cloquintocet-mexyl are similar those observed in previous research when topramezone was applied at 37 g ha^-1^ [15]. Topramezone caused less injury in plants treated with cloquintocet-mexyl + malathion (46%) than with malathion alone (79%). Malathion, ABT, cloquintocet-mexyl, or combinations thereof did not cause any visible injury in the absence of topramezone (data not presented).

### Biomass responses 10 days after treatment

Leaf biomass of plants treated with topramezone alone measured 58% of the non-treated control (Fig 3). Compared to topramezone alone, biomass was lower in plants treated with malathion + topramezone (38% of non-treated control). Biomass of plants treated with cloquintocet-mexyl or cloquintocet-mexyl + ABT, were not different from those treated with topramezone alone. When applied without topramezone, malathion, ABT, cloquintocet-mexyl, or combinations thereof did not cause any leaf biomass reductions compared to the non-treated control, which supports visual injury responses (data not presented).

Creeping bentgrass biomass responses to certain treatments were not consistent with visible responses. Similar inconsistencies were observed in our previous research that also evaluated sub-lethal rates of topramezone in creeping bentgrass [15]. A potential explanation is that sub-lethal applications of HPPD-inhibitors cause leaf bleaching without immediate reductions in biomass. Biomass responses more consistent with visible responses may have been observed in more immature creeping bentgrass plants with less recuperative capacity. However, creeping bentgrass is grown as a perennial turfgrass, so understanding the effects of P450 monooxygenase inhibitors on immature creeping bentgrass tolerance to topramezone would have fewer practical implications.

## Discussion

Reductions in leaf tissue biomass, concomitant with increases in visible bleaching and necrosis caused by malathion, suggests that P450 monooxygenase function influences topramezone metabolism in creeping bentgrass. Visible responses demonstrate malathion is a more potent synergist of topramezone in creeping bentgrass than ABT. Previous research investigating metabolism-based chlorsulfuron resistance in *Lolium rigidum* also found that malathion increased chlorsulfuron toxicity more than ABT [42]. Cloquintocet-mexyl completely eliminated creeping bentgrass visible injury from topramezone, supporting responses observed in our previous research [15]. Cloquintocet-mexyl antagonized the effects of malathion or ABT on topramezone, but the implications of this are not clear. It is possible that cloquintocet-mexyl increased topramezone metabolism through mechanisms not catalyzed by P450 monooxygenases, as cloquintocet-mexyl is known to increase pyridinyl O-glucosylation in wheat [28]. We speculate that topramezone could be O-glucosylated in creeping bentgrass. However, it is more probable that P450 monooxgenase activity was higher in plants treated with cloquintocet-mexyl even in the presence of ABT or malathion, or that certain P450’s induced were not inhibited by malathion and ABT. Yun et al. [48] evaluated the effects of the P450 monoxygenase inhibitor PBO alone and in combination with safeners on the activity of microsomal pyrazosulfuron-ethyl *O*-demethylase, which metabolizes pyrazosulfuron-ethyl in rice (*Oryza sativa* L.). They found that PBO inhibited 63% of pyrazosulfuron-ethyl *O*-demethylase activity when in combination with the herbicide, but only 19% inhibition was achieved when PBO was in combination with the herbicide and the safener. The researchers suggested that this was because the herbicide and safeners induce different P450 monooxygenases. Previous research indicates that ABT inhibits P450 monooxygenases responsible for aryl ring hydroxylation but not those responsible for N-demethylation [32,34].

Future research should determine the contribution of cytochrome P450 monooxygenase and GST activity on topramezone metabolism in creeping bentgrass. This could involve using mass spectroscopy to elucidate topramezone metabolite formation in safened and non-safened creeping bentgrass plants, which would suggest P450 or GST involvement. It will be important to determine whether there are additional primary routes of metabolism beyond the N-demethylation proposed by previous research [9]. Another method could involve *in vitro* GST and cytochrome P450 enzyme assays with safened and non-safened creeping bentgrass extracts.
